# Inhibition of Poly(ADP-ribose)polymerase impairs Epstein Barr Virus lytic cycle progression

**DOI:** 10.1186/1750-9378-2-18

**Published:** 2007-10-11

**Authors:** Stefania Mattiussi, Italo Tempera, Giulia Matusali, Giulia Mearini, Luisa Lenti, Silvia Fratarcangeli, Luciana Mosca, Maria D'Erme, Elena Mattia

**Affiliations:** 1 Dept. of Biochemical Sciences, University "Sapienza", P.le Aldo Moro, 5, 00185, Rome, Italy; 2 Dept. of Public Health Sciences, University "Sapienza", P.le Aldo Moro, 5, 00185, Rome, Italy; 3 Dept. of Experimental Medicine, University "Sapienza", V.le Regina Elena 324, 00161 Rome, Italy

## Abstract

**Background:**

Poly(ADP-ribosylation) is a post-translational modification of nuclear proteins involved in several cellular events as well as in processes that characterize the infective cycle of some viruses. In the present study, we investigated the role of poly(ADP-ribosylation) on Epstein-Barr Virus (EBV) lytic cycle activation.

**Results:**

Inhibition of PARP-1 by 3-aminobenzamide (3-ABA) during EBV induction, diminished cell damage and apoptosis in the non-productive Raji cell line while markedly reducing the release of viral particles in the productive Jijoye cells. Furthermore, incubation with 3-ABA up-regulated the levels of LMP1 and EBNA2 latent viral proteins. At the same time, it slightly affected the expression of the immediate early BZLF1 gene, but largely down-regulated the levels of the early BFRF1 protein. The modulation of the expression of both latent and lytic EBV genes appeared to be post-transcriptionally regulated.

**Conclusion:**

Taken together the data indicate that PARP-1 plays a role in the progression of EBV lytic cycle and therefore, PARP inhibitors might represent suitable pharmacological adjuncts to control viral spread in EBV productive infection.

## Background

Epstein Barr Virus (EBV), the ethiological agent of infectious mononucleosis (IM) is associated with a number of tumors such as Burkitt's lymphoma (BL), Hodgkin's disease (HD), nasopharingeal carcinoma (NPC) and with lymphoproliferative diseases in the immunocompromised individuals [1]. The virus has two distinct cycles of infection: latent and lytic. During latency, a limited number of genes is differentially expressed. These include six nuclear antigens, designated as EBNA-1 to -6, three membrane proteins, indicated as LMP-1, -2A, and -2B and two small non-polyadenylated RNAs (EBERs).

EBV nuclear antigen EBNA1 is required for latent replication, episomal mainteinance and viral genome segregation [2]. EBNA2, EBNA-3A, -3B and -3C are transcriptional activators of viral and cellular genes. With the exception of EBNA-3B, they all concurr with the EBERs to B cell transformation [3].

Among the latent genes, LMP-1 is essential for B-lymphocyte transformation. It upregulates anti-apoptotic genes such as Bcl-2 and Mcl-1 [4], induces several cell surface adhesion molecules and activation markers and stimulates cytokine production [5].

During the lytic cycle, the sequential expression of immediate early, early and late genes, leads to production of viral particles. The EBV lytic cycle cascade initiates with the expression of two immediate-early genes: BZLF1 encoding for ZEBRA, and BRLF1 encoding for Rta. The two viral products promote each other expression, transactivate separate classes of EBV lytic genes and together coordinate the activation of a third class of lytic genes [1].

In vivo, reactivation of the virus occurs in terminally differentiated plasma cells in response to antigen stimulation [6]. In vitro, the lytic cycle can be induced by different agents, such as phorbol esters, sodium butyrate, antiimmunoglobulins (anti-IgG) and calcium ionophores [7-9].

Although many studies have been devoted to elucidate the molecular events underlying EBV activation, the role that epigenetic modifications play in this process, is still unclear. In this respect, histone acetylation as well as DNA methylation of the BZLF1 promoter (Zp) have been shown to occur in the transition from the latent to lytic phase [10].

Poly(ADP-ribosylation) is a post-translational modification of nuclear proteins that appears to be involved in several cellular events such as DNA repair, cell differentiation, apoptosis and tumor promotion [11].

The poly(ADP-ribose)polymerase (PARP-1), a zinc-binding nuclear enzyme, catalyzes the covalent addition of the ADP-ribose moiety of nicotinamide adenine dinucleotide (NAD^+^) to nuclear proteins including histones, transcription factors and PARP itself as well as the subsequent elongation step of the polymer. Because of its negative charges, the poly(ADP-ribose)polymer highly affects the function of target proteins [12]. Moreover, also non-covalently bound poly(ADP-ribose)polymers have been shown to modulate the activity of several proteins [13].

PARP-1 is required during transcriptional activation of Drosophila puff loci [14], it is a structural component of chromatin in polytene chromosome [15] and modulates the activity of transcription factors [16].

It has been shown that poly(ADP-ribosylation) is needed for fundamental events that characterize the infective cycle of several viruses. In fact this process is involved in the regulation of the replication and transcription activator (RTA) of gamma-2 herpesvirus [17], in the replication and integration of HIV-1 [18,19], while it contributes to decapsidation of adenovirus [20] and papillomavirus [21]. In addition, recent data indicate that macro domains of some RNA viruses bind efficiently free and automodified PARP-1, possibly modulating the host response to viral infection [22].

In this study we have examined the role that poly(ADP-ribosylation) plays in the EBV activation process by inducing the lytic cycle in the presence of 3-aminobenzamide (3-ABA), a well known inhibitor of PARP-1 activity [23].

To this end we have treated Burkitt lymphoma-derived Raji and Jijoye cells with agents able to induce EBV lytic cycle. However, a deletion of EBV genome in Raji cells prevents late gene expression, leading to an abortive cycle [24], while the complete productive infection is supported by Jijoye cells.

It has been shown that 3-ABA might also inhibit cell death and apoptosis [25], possibly by interfering with cytoskeleton organization [26] and/or cell-cycle checkpoint mechanisms [27].

We report here that treatment of Burkitt's lymphoma cells with 3-ABA, besides exerting cytoprotective and antiapoptotic effects, modulates the expression of both latent and lytic EBV genes impairing viral lytic cycle progression and particles release.

## Results

### Effect of 3-ABA on EBV lytic cycle activation and apoptosis

As previously reported, exposure of Raji cells to P(BU)_2_, sodium butyrate and TGF-β2, induces EBV lytic cycle in about 70% of the population, as judged by immunofluorescence detection of EA expression [28].

**Figure 1:**
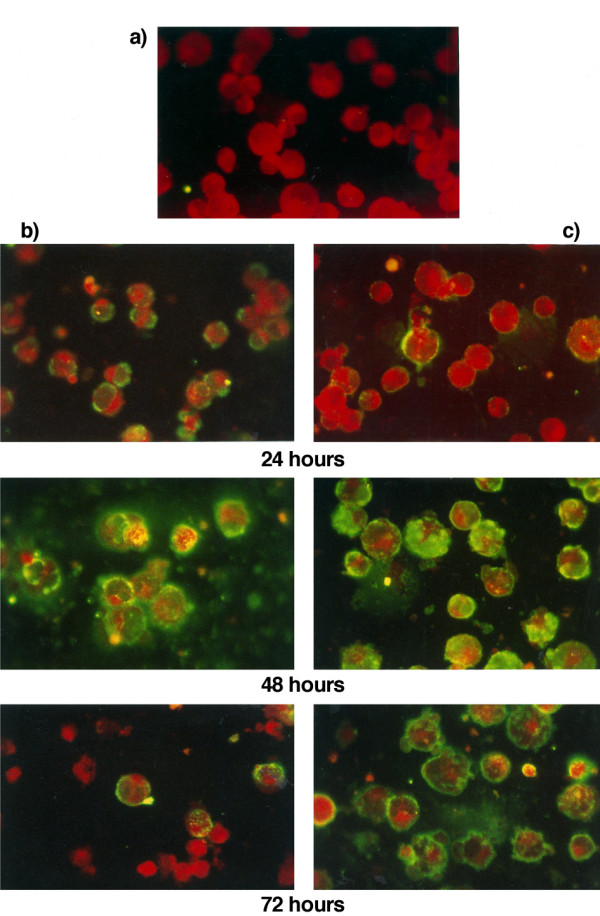
**Representative microphotographs showing early antigen expression in Raji cells induced with or without 3-ABA**. EBV lytic cycle was induced as described in the Methods in the absence (b), or in the presence (c) of 3 mM 3-ABA. Latently infected Raji cells exposed or not to 3-ABA (a) were used as control. At the indicated times (24, 48 and 72 hours) cells were collected and stained with FITC-labeled EA antibodies and Blue Evans dye.

The effect of PARP inhibition on EBV early antigen expression was therefore evaluated on Raji cells induced in the presence or in the absence of 3-ABA and on latently-infected Raji cells used as controls. Fig. 1 shows representative microphotographs obtained from cells collected at different times during incubation. The image in a) shows control cells, negative for EA staining, independently of the incubation with the PARP-1 inhibitor. The pictures reported in column b) show that after 24 hours incubation with lytic cycle inducers, FITC-labeled antibodies detected EA in a large percentage of the cell population, and the intensity of the fluorescence increased at 48 hours. In comparison, the expression of EA in the presence of 3-ABA (panel c) occurred in a lower number of cells at 24 hours while at 48 hours the percentage of positive cells and the intensity of the signal seemed comparable or even higher than what reported in panel b).

At 72 hours, in the absence of 3-ABA, along with positively stained cells, a number of damaged cells, smaller than intact ones, could be visualized. In contrast, Raji cells incubated with PARP-1 inhibitor were stained similarly to the samples incubated for 48 hours, with the signal mainly localized at the periphery of the cells.

cytofluorymetric analysis of cells treated as for the IF studies, indicated that the percentage of the cell population undergoing apoptosis (pre-G1 peak) was slightly affected by the presence of the PARP-1 inhibitor within the first 48 hours of incubation. However, at 72 hours, a 34% of apoptotic cells was observed in induced Raji cells, while only a 20% was measured when lytic cycle activation was carried out in the presence of 3-ABA (data not shown).

**Figure 2:**
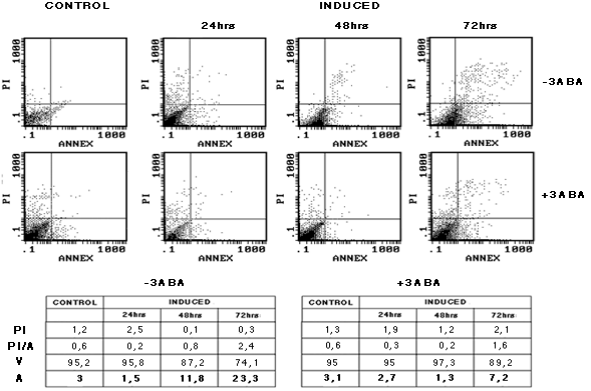
**Cytofluorymetric analysis of apoptotic Raji cells**. Raji cells were induced in the absence or in the presence of 3-ABA as for Fig.1. At the indicated times, cells were collected and subjected to Annexin V-FITC/PI staining as reported in the Methods. The upper part of the figure shows the dot blots of PI vs. Annexin V stain. All parameters and region settings were kept identical throughout all measurements. The table illustrates the percentages of necrotic cells (PI); secondary necrotic cells (PI/A); viable cells (V); Annexin V-positive (apoptotic) cells (A).

To further investigate the protective effect of 3-ABA during EBV lytic cycle activation the rate of apoptosis was tested by Annexin V assay. Fig. 2 shows the PI versus Annexin V dot plot of a representative experiment performed on Raji cells treated as above described. The data of the cytofluorymetric analysis indicate that after 48 and 72 hours of exposure to the lytic cycle inducing compounds, cell viability was reduced to about 87 and 74%, respectively, while the corresponding measurements of the cells exposed to 3-ABA were 97 and 89%. At the same time points, the percentages of Annexin V-positive cells were 11 and 23%, respectively. However, the fraction of apoptotic cells was markedly lower (1.3 and 7.2%) in the samples incubated in the presence of 3-ABA.

### Effect of 3-ABA on the early and on late phases of EBV lytic cycle

**Figure 3:**
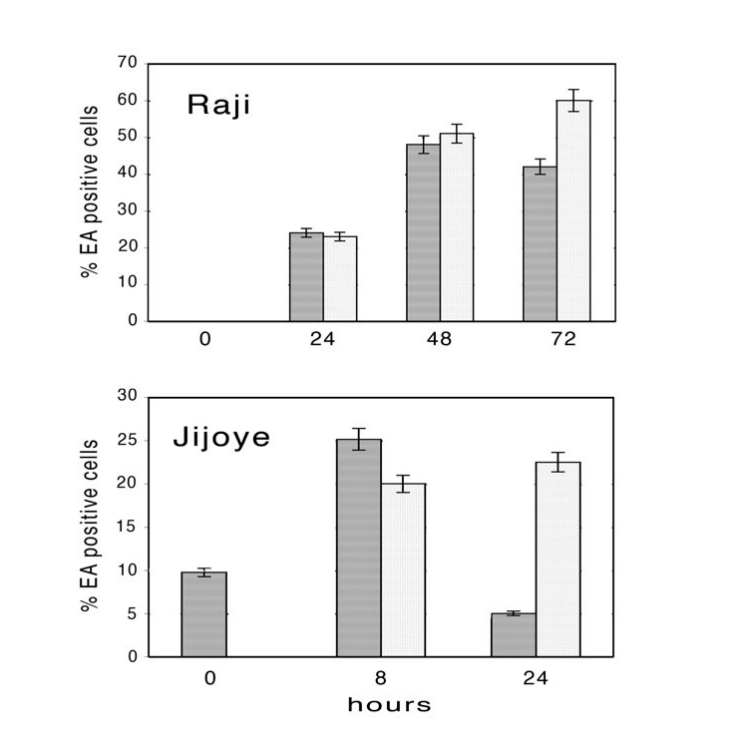
**Cytoflowrimetric analysis of EA expression in Raji and Jijoye cells**. EBV lytic cycle was induced in Raji and Jijoye cells in the absence or in the presence of 3-ABA as described in the Methods. At the indicated times, cels were analyzed by flowcytometry after staining with FITC-EA antibodies. The bargraph represents the percentages of EA-positive cells determined after induction in the absence (dark bars) or in the presence (light bars) of 3-ABA. Error bars are the means ± SD of three independent experiments.

To evaluate the effect of 3-ABA on the early phases of EBV lytic cycle, the percentage of EA-positive cells was determined by cytofluorymetric analysis in Raji and Jijoye cells incubated with lytic cycle activators in the absence or in the presence of the PARP inhibitor. The results reported in the upper panel of Fig 3 show that in Raji cells, the percentage of the population expressing the EA after 24 and 48 hours of induction was not significantly altered by the presence of 3-ABA, representing about 20 and 50%, respectively. In contrast, after 72 hour of incubation with the inducers, about 20% increment of EA-positive cells was observed when the PARP inhibitor was added to the colture.

The lower panel of Fig. 3 illustrates the results obtained from Jijoye cells. Since in this cell line, EBV supports the complete lytic cycle, samples were collected up to 24 hours, when most of the cells were intact. The data reported show that spontaneous lytic cycle activation occurred in the absence of the inducing compounds in about 10% of the untreated cells. Eight hours after the addition of the inducers, about 25% of Jijoye cells became positive for the EA; this fraction was slightly lower when the cells were induced in the presence of the PARP inhibitor. In contrast, after 24 hours, more than 20% of the cells incubated with 3-ABA were still expressing the EA, while this fraction was reduced to 5% in the absence of the inhibitor.

In order to assess whether the higher percentage of EA-positive cells in the samples treated with 3-ABA for 24 hours reflected an impairment in EBV lytic cycle completion, the release of viral DNA in the colture medium, was determined.

**Figure 4:**
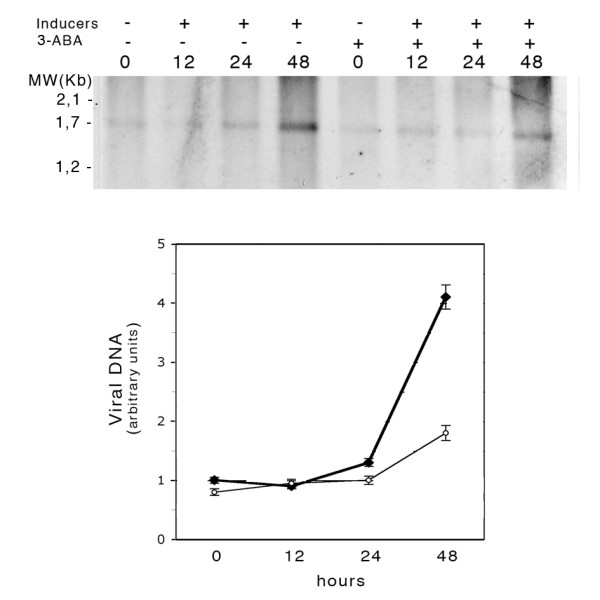
**Detection of EBV DNA released in the colture medium of induced Jijoye cells**. Jijoye cells were induced with or without 3-ABA as described in the Methods. Viral DNA purified from the colture medium was digested with BamH1 and analyzed by Southern blot with a DIG-tailed probe specific for the BamH1 Z region of the EBV genome. The values in the graph were obtained by densitometric measurements of the 1700 bp BamH1 Z fragment of the viral DNA collected in the supernatant of Jijoye cells induced in the absence (filled circles) or in the presence (open circles) of 3-ABA.

Fig. 4 shows the results of the hybridization of BamH1-digested EBV DNA obtained from the supernatant of cells induced for different lengths of time in the presence or in the absence of 3-ABA, with a probe for the BamH1 Z region of the EBV genome. Quantification of the signal corresponding to the 1700 bp specific fragment revealed that at 48 hours, in the presence of the PARP inhibitor, viral DNA released in the supernatant was about half the amount detected in the absence of 3-ABA.

### Effect of PARP-1 inhibitor on EBV protein levels

To evaluate the effect of PARP-1 inhibition on EBV gene expression, the levels of two latent (LMP1 and EBNA-2), and two lytic (BZLF1 and BFRF1) viral proteins, were analyzed by Western blot.

**Figure 5:**
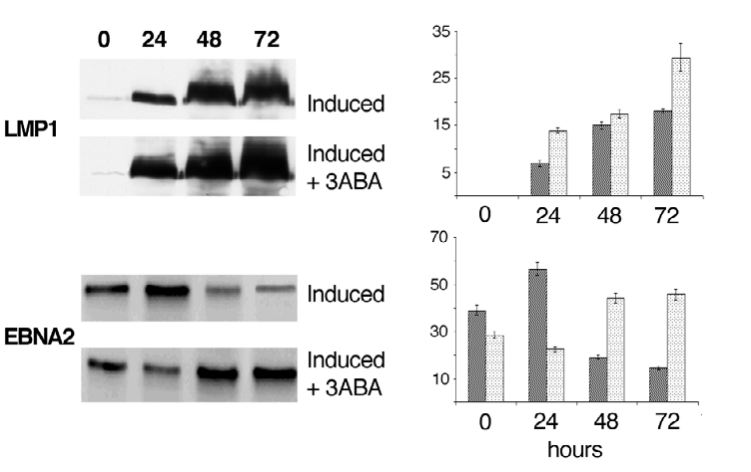
**LMP1 and EBNA2 expression by Western blot analysis**. Cell lysates obtained from Raji cells treated and collected as reported in Fig.1, were subjected to SDS-PAGE. The blots were probed with LMP-1 and EBNA2 antibodies. Specific signals, quantified by densitometry are expressed as arbitrary units in the bargraph. Error bars are the means ± SD of three independent experiments. Dark bars: induced Raji cells; light bars: induced Raji cells + 3-ABA.

Fig. 5 shows LMP1 and EBNA2 expression in induced Raji cells incubated for different times with or without 3-ABA. In the upper panel it appears that LMP1 levels strongly increased during incubation with EBV lytic cycle inducers. However, the increment was larger in the cells induced in the presence of the PARP-1 inhibitor. In particular, in the latter, at 72 hours, the level of the protein was about 1.6 fold higher than that measured in the absence of 3-ABA. In the lower panel, the results of a representative blot hybridized with EBNA2 antibodies are shown. Similarly to what observed for LMP1, EBNA2 levels in the cells induced for 48 and 72 hours in the presence of 3-ABA appeared about 2.5 fold higher than those measured in the absence of the inhibitor. Similar results were obtained by using the structurally unrelated PARP-1 inhibitor PJ 34.

**Figure 6:**
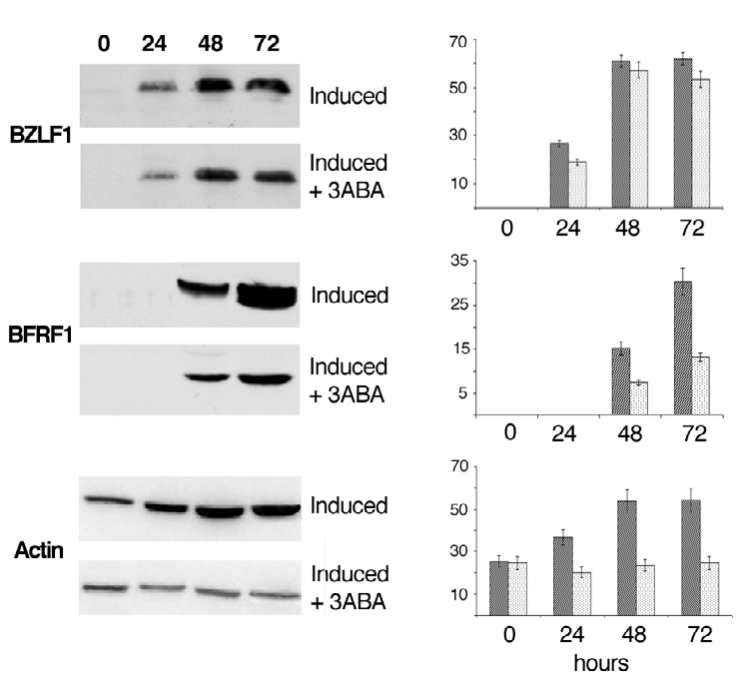
**BZLF1 and BFRF1 expression by Western blot analysis**. Cell lysates obtained from Raji cells treated and collected as reported in Fig.1, were subjected to SDS-PAGE. The blots were probed with BZLF1, BFRF1 and actin antibodies. Specific signals, quantified by densitometry are expressed as arbitrary units in the bargraph. Error bars are the means ± SD of three independent experiments. Dark bars: induced Raji cells; light bars: induced Raji cells + 3-ABA.

Fig. 6 illustrates the results obtained when the blots were tested with antibodies for BZLF1 or BFRF1, an immediate early and an early EBV lytic gene, respectively. It is shown that BZLF1 expression gradually increased during the first 48 hours that followed the addition of lytic cycle activators to Raji cells. In the presence of 3-ABA the extent of this increment was slightly lower than that observed in the absence of the inhibitor. The middle panel shows that the signal identifying BFRF1 appeared 48 hours after EBV induction and increased of about two fold at 72 hours. Remarkably, the increment of BFRF1 protein in Raji cells induced in the presence of 3-ABA was about 50% lower than that measured for both time points in the absence of the inhibitor.

To verify that equal amounts of cellular proteins had been loaded on gels, blots were hybridized with actin antibodies. Surprisingly, incubation of Raji cells with EBV lytic cycle inducers led to a 2 fold increment of actin levels. This increment was largely prevented when EBV induction was carried out in the presence of 3-ABA.

Antibodies directed to glyceraldehyde-3-phosphate dehydrogenase (GAPDH) were used to verify that similar amounts of proteins had been loaded onto each gel track (data not shown).

The effect of PARP inhibitor on EBV latent and lytic gene expression was also evaluated in Jijoye cells treated with lytic cycle inducing agents. The results obtained with this cell line were substantially similar to those above described for Raji cells (data not shown).

### Effect of PARP-1 inhibitor on EBV mRNA levels

**Figure 7:**
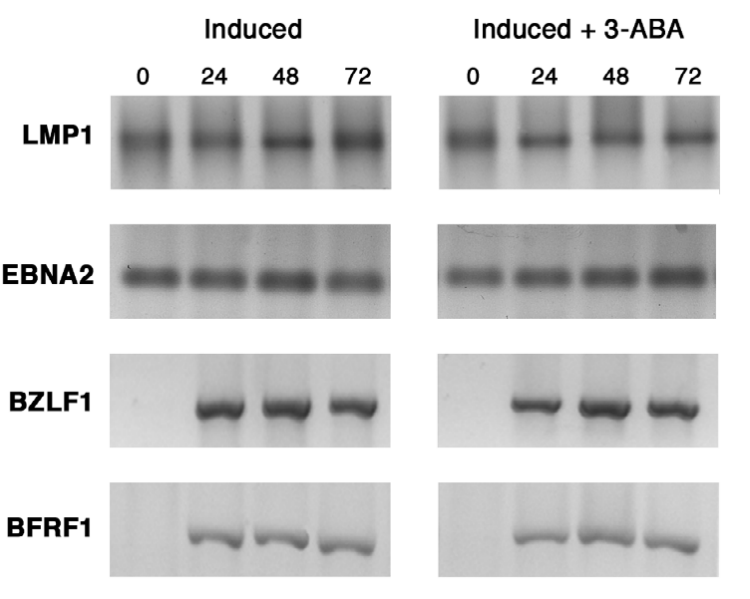
**Expression of EBV latent and lytic genes by RT-PCR**. Total RNA was purified from Raji cells induced in the absence or in the presence of 3-ABA and collected at the indicated times. mRNA levels of LMP-1, EBNA2, BZLF1 and BFRF1 were determined by RT-PCR as described in the Methods. Amplification products were separated on a 1.5% agarose gel and stained with ethidium bromide.

To assess whether the differences observed in the levels of EBV latent and lytic products resulted from changes in the rate of transcription of the corresponding genes, mRNA levels were measured by RT-PCR. Fig. 7 illustrates the results obtained with primers for LMP1, EBNA2, BZLF1 and BFRF1 respectively. It appears that for the two latent genes, as well as for the two lytic genes, the rates of transcription were not significantly affected by the addition of 3-ABA during EBV induction.

Control experiments performed with the primers for the GAPDH gene, confirmed that equal amounts of total RNA had been subjected to RT-PCR (data not shown).

## Discussion

The present study investigated the role that poly(ADP-ribosylation) plays during EBV lytic cycle activation in Burkitt's lymphoma-derived Raji and Jijoye cells, the first supporting only the early phases of the lytic cycle, the latter allowing the complete, productive infection. We report here that upon induction of EBV lytic cycle in the presence of 3-ABA, high levels of EA were detected in both cell lines even at late times of incubation while a reduced amount of viral DNA was measured in the colture medium of the productive Jijoye cells. These data strongly suggest that the PARP inhibitor affects the progression and the completion of EBV lytic cycle.

Moreover, our observations indicate that incubation with 3-ABA during EBV induction favors the maintenance of Raji cell integrity and reduces the apoptotic events.

It has been reported that activators of EBV lytic program induce apoptosis, but that lytic gene expression protects from cell death [29]. Nevertheless, in Raji cells where the lytic cycle is blocked at the early phases, a prolonged incubation with the inducers, resulted in about 20% of apoptosis. The large reduction of apoptotic cells detected in the presence of 3-ABA confirms the cytoprotective effect that has been reported for the PARP-1 inhibitor in different experimental model of apoptosis [30,27].

Besides, PARP-1 inhibition alters the expression of two latent and two lytic viral proteins. The observed up-regulation of the latent LMP1 and EBNA2 and the down-regulation of the lytic BZLF1 and BFRF1 proteins, concur with impairment of the viral activation process.

Because the up-regulation of LMP1 and EBNA2 expression seems to take place at the post-transcriptional level it is reasonable to hypothesize a lower rate of turnover for the two viral products. Little is known about the degradation pathway of EBNA 2. However, it has been shown that the turnover of LMP-1, ubiquitinated in the short cytoplasmic N-terminus, occurs *via *the proteasome [31]. Since PARP-1 can stimulate proteasome activity by the addition of ADP polymers to the 20S proteasome subunit [32], the increment of LMP1 protein, detected in induced Raji cells exposed to 3-ABA might result from a lower activity of the proteasomal complex.

In agreement with the stimulatory effect of LMP1 on Bcl2 expression [4], the higher levels of the antiapoptotic protein detected in 3-ABA treated cells (data not shown) might also contribute to the cyto-protective effect observed with PARP-1 inhibitor.

Our data indicate that actin levels rise in the presence of EBV lytic cycle inducers. This event is possibly related to actin remodeling by phorbol esthers [33] and/or by LMP1 activation of Rho GTPase [34]. The addition of PARP-1 inhibitor to induced Raji cells, largely prevents actin increment. Several reports indicate that 3-ABA activity is associated with cell type specific cytoscheleton rearrangements, mostly due to modification of actin polymerization or assembly [26,35]. Our results lead to hypotesize that 3-ABA treatment might, in addition, interfere with the mechanisms that regulate actin intracellular levels.

The present work demonstrates that the increment of the early EBV protein BFRF1, was dramatically inhibited when EBV lytic cycle was induced in the presence of 3-ABA while only a slight decrement was observed for BZLF1 protein. These observations indicate that PARP-1 inhibition does not affect the switch from the latent to the lytic cycle of EBV infection, but rather the following event/s leading to the sequential expression of EBV lytic genes cascade. In this respect, PARP-1 inhibition could contribute to determine an abortive lytic cycle, a phenomenon that might be relevant in the early phases of B lymphocytes infection.

Because PARP-1 is involved in decondensation of high-order chromatin structure [12,36], inhibition of the enzyme might determine a lower accessibility of DNA to the transcription machinery. Moreover, PARP-1 activity is required to repair DNA strand break. We do not know whether the inhibition of this function might impair the successful transcription of some EBV lytic genes. Our data however, indicating that the rates of transcription of BFRF1 are not influenced by the 3-ABA, suggest that the effect of 3-ABA on BFRF1 protein levels is either indirect or mainly exerted at the post-transcriptional level.

## Conclusion

The data reported in the present study indicate that PARP-1 inhibition impairs EBV lytic cycle progression, thereby affecting the release of viral particles.

We envisage that these results might lead to consider PARP-1 inhibitors as potential therapeutic agents to control the spread of EBV productive infection.

## Methods

### Cell culture conditions

Burkitt's lymphoma derived Raji and Jijoye cells were grown in RPMI 1640 medium (Sigma) supplemented with 1% penicillin-streptomycin and 5% fetal calf serum (FCS) in a 5% CO_2 _atmosphere.

### Induction of EBV lytic cycle and treatment with 3-ABA

EBV lytic cycle was induced in Raji cells as previously described [28] in the presence or in the absence of 3 mM 3-ABA. Control experiments were carried out with latently-infected cells, incubated with 3 mM 3-ABA. At 0, 24, 48 and 72 hours, cell samples were collected and analysed as described below. Cell viability was assessed by trypan blue exclusion.

### Fluorescence microscopy

Treated cells were smeared on slide, fixed and permeabilized with methanol:acetone (2:1) at -20°C and then rehydrated with phosphate buffered saline solution (PBS), pH 7.4. Fixed cells were incubated with FITC-conjugated F_6_-Ester 2 antibodies [37] recognizing EBV early antigens (EA) diluted 1:40 in PBS/1% BSA, for 1 hour at 37°C. After three washes with PBS, cells were stained with 100 μg/ml Blue Evans dye for 5 min at room temperature. After three more washes, slides were mounted with 50% glycerol in PBS and analyzed with a Leitz Orthoplan immunofluorescence microscope. Images were recorded by an Olympus digital camera.

### Cytofluorymetric determinations

Cells incubated with EBV lytic cycle inducers in the absence or in the presence of 3-ABA were collected at different times. The distribution of Raji cells in the cell cycle phases was determined by FACS analysis after DNA staining with propidium iodide (PI). Cell samples were washed with PBS and centrifuged for 5 min at 400 x g. The cell pellet was incubated for 1 h at 4°C in 70% ethanol, washed again with PBS, and finally stained in the dark for 1 hour with 100 μg/ml of PI and 100 μg/ml RNase in PBS. pre-G1 peak, characterized by the lowest PI staining intensity, mainly represented apoptotic cells.

To evaluate apoptosis with an additional method, cells treated and collected as above described were resuspended in ice-cold binding buffer and stained by Annexin V-FITC Kit (Beckman Coulter) according to the accompanying procedure.

To measure EA expression, cells incubated with EBV lytic cycle inducers in the absence or in the presence of 3-ABA were collected at different times, washed with PBS and fixed in ice cold PBS containing 2% paraformaldehyde for 20 min. After two more washes in PBS, the cells were resuspended in 60 μM digitonin containing FITC-conjugated F_6_-Ester 2 antibodies diluted 1:20 and incubated for 60 min at 37°C.

All cytofluorymetric determinations were carried out by Coulter EPICS XL.

### Detection of EBV genome released in the supernatant

Jijoye cells (1.5 x 10^6^) were exposed to lytic cycle inducing agents with or without 3-ABA, for 12, 24 and 48 hours. Colture supernatants were concentrated in Vivaspin 2, 10000 MWCO HY (Sartorius) by a centrifugation in swing rotor for 5 min at 4000 x g. Viral DNA was purified from the concentrated samples by QIAamp DNA Mini kit according to the manufacturer instruction and subjected to BamH1 digestion. DNA (10 μl) was resolved by electrophoresis on a 0.7% agarose gel and blotted on Nylon membrane (Roche Diagnostic). Hybridization was carried out in DIG Easy granules solution (Roche Diagnostic) with 50 ng/ml of a Digoxigenin-tailed DNA probe recognizing the BamH1 Z fragment of the EBV genome [28]. Specific signals were quantified by densitometric analysis (ImageJ freeshare software).

### Western blot analysis

At the indicated times, samples (about 10^6 ^cells) were collected, washed and lysed as described [38]. 30 μg of proteins, as determined by a modified Lowry assay (RC DC protein assay, BioRad), were resolved by SDS-PAGE on a 10% gel and transferred onto nitrocellulose membrane. Primary antibodies used: LMP-1 (BD PharMingen), EBNA2 (kind gift of Prof. M. Rowe), BZLF1 (Argene Biosoft), BFRF1 (kindly provided by Dr. A. Farina), β-actin (Sigma), and Bcl-2 (Santa Cruz). Specific signals visualized by ECL detection kit (Amersham Pharmacia Biotech) were quantified by densitometric analysis (ImageJ freeshare software).

### RT-PCR experiments

Isolation of total RNA, primers sequences and the size of amplification products of LMP1, GAPDH and EBNA2, were as previously reported [39]. Primers used for BZLF1 (DP 5'-TTCAAAGAGAGCCGACAGGA-3'; RP 5'-ATCGCAAGCTCCTTTGCCT-3') and for BFRF1 (DP 5'-CCTAGATCTAGTGAATCATG-3'; RP 5'-TTCTGAAAAGTTATCCAAGT-3') amplified a product of 704 and 730 bp, respectively. PCR products were loaded onto a 1.5% agarose gels with 0.5 μg/ml ethidium bromide and visualized under UV light.

## Competing interests

The author(s) declare that they have no competing interests.

## Authors' contributions

SM and IT equally contributed to the present work. They carried out most of the experimental work and contributed to draft the manuscript. GMa, GMe and SF accomplished cell colture treatments and helped to elaboration of data. LL carried out cytofluorymetric analysis. LM contributed to elaboration of data and helped to draft the manuscript. MD conceived of the study, participated in its design and helped to draft the manuscript. EM participated and coordinated the study, compiled and finalized the manuscript. All authors read and approved the final manuscript.

## References

[B1] Rickinson AB, KE B (2001). Epstein Barr Virus.

[B2] Lee MA, Diamond ME, Yates JL (1999). Genetic evidence that EBNA-1 is needed for efficient, stable latent infection by Epstein-Barr virus. J Virol.

[B3] Tomkinson B, Robertson E, Kieff E (1993). Epstein-Barr virus nuclear proteins EBNA-3A and EBNA-3C are essential for B-lymphocyte growth transformation. J Virol.

[B4] Henderson S, Rowe M, Gregory C, Croom-Carter D, Wang F, Longnecker R, Kieff E, Rickinson A (1991). Induction of bcl-2 expression by Epstein-Barr virus latent membrane protein 1 protects infected B cells from programmed cell death. Cell.

[B5] Eliopoulos AG, Gallagher NJ, Blake SM, Dawson CW, Young LS (1999). Activation of the p38 mitogen-activated protein kinase pathway by Epstein-Barr virus-encoded latent membrane protein 1 coregulates interleukin-6 and interleukin-8 production. J Biol Chem.

[B6] Thorley-Lawson DA (2001). Epstein-Barr virus: exploiting the immune system. Nat Rev Immunol.

[B7] Di Renzo L, Avila-Carino J, Klein E (1993). Induction of the lytic viral cycle in Epstein Barr virus carrying Burkitt lymphoma lines is accompanied by increased expression of major histocompatibility complex molecules. Immunol Lett.

[B8] Luka J, Kallin B, Klein G (1979). Induction of the Epstein-Barr virus (EBV) cycle in latently infected cells by n-butyrate. Virology.

[B9] Takada K, Ono Y (1989). Synchronous and sequential activation of latently infected Epstein-Barr virus genomes. J Virol.

[B10] Jenkins PJ, Binne UK, Farrell PJ (2000). Histone acetylation and reactivation of Epstein-Barr virus from latency. J Virol.

[B11] Burkle A (2001). Physiology and pathophysiology of poly(ADP-ribosyl)ation. Bioessays.

[B12] d'Erme M, Yang G, Sheagly E, Palitti F, Bustamante C (2001). Effect of poly(ADP-ribosyl)ation and Mg2+ ions on chromatin structure revealed by scanning force microscopy. Biochemistry.

[B13] Althaus FR (2005). Poly(ADP-ribose): a co-regulator of DNA methylation?. Oncogene.

[B14] Tulin A, Spradling A (2003). Chromatin loosening by poly(ADP)-ribose polymerase (PARP) at Drosophila puff loci. Science.

[B15] Kim MY, Mauro S, Gevry N, Lis JT, Kraus WL (2004). NAD+-dependent modulation of chromatin structure and transcription by nucleosome binding properties of PARP-1. Cell.

[B16] Oei SL, Griesenbeck J, Schweiger M, Ziegler M (1998). Regulation of RNA polymerase II-dependent transcription by poly(ADP-ribosyl)ation of transcription factors. J Biol Chem.

[B17] Gwack Y, Nakamura H, Lee SH, Souvlis J, Yustein JT, Gygi S, Kung HJ, Jung JU (2003). Poly(ADP-ribose) polymerase 1 and Ste20-like kinase hKFC act as transcriptional repressors for gamma-2 herpesvirus lytic replication. Mol Cell Biol.

[B18] Ha HC, Juluri K, Zhou Y, Leung S, Hermankova M, Snyder SH (2001). Poly(ADP-ribose) polymerase-1 is required for efficient HIV-1 integration. Proc Natl Acad Sci USA.

[B19] Kameoka M, Nukuzuma S, Itaya A, Tanaka Y, Ota K, Ikuta K, Yoshihara K (2004). RNA interference directed against Poly(ADP-Ribose) polymerase 1 efficiently suppresses human immunodeficiency virus type 1 replication in human cells. J Virol.

[B20] Dery CV, de Murcia G, Lamarre D, Morin N, Poirier GG, Weber J (1986). Possible role of ADP-ribosylation of adenovirus core proteins in virus infection. Virus Res.

[B21] Carbone M, Reale A, Di Sauro A, Sthandier O, Garcia MI, Maione R, Caiafa P, Amati P (2006). PARP-1 interaction with VP1 capsid protein regulates polyomavirus early gene expression. J Mol Biol.

[B22] Egloff MP, Malet H, Putics A, Heinonen M, Dutartre H, Frangeul A, Gruez A, Campanacci V, Cambillau C, Ziebuhr J (2006). Structural and functional basis for ADP-ribose and poly(ADP-ribose) binding by viral macro domains. J Virol.

[B23] Rankin PW, Jacobson EL, Benjamin RC, Moss J, Jacobson MK (1989). Quantitative studies of inhibitors of ADP-ribosylation in vitro and in vivo. J Biol Chem.

[B24] Hatfull G, Bankier AT, Barrell BG, Farrell PJ (1988). Sequence analysis of Raji Epstein-Barr virus DNA. Virology.

[B25] Hassa PO, Haenni SS, Elser M, Hottiger MO (2006). Nuclear ADP-ribosylation reactions in mammalian cells: where are we today and where are we going?. Microbiol Mol Biol Rev.

[B26] Malorni W, Rainaldi G, Straface E, Rivabene R, Cossarizza A, Capri M, Monti D, Franceschi C (1994). 3-Aminobenzamide induces cytoskeleton rearrangement in M14 melanoma cells. Biochem Biophys Res Commun.

[B27] Masutani M, Nozaki T, Wakabayashi K, Sugimura T (1995). Role of poly(ADP-ribose) polymerase in cell-cycle checkpoint mechanisms following gamma-irradiation. Biochimie.

[B28] Mattia E, Ceridono M, Chichiarelli S, D'Erme M (1999). Interactions of Epstein-Barr Virus Origins of Replication with Nuclear Matrix in the Latent and in the Lytic Phases of Viral Infection. Virology.

[B29] Inman GJ, Binne UK, Parker GA, Farrell PJ, Allday MJ (2001). Activators of the Epstein-Barr virus lytic program concomitantly induce apoptosis, but lytic gene expression protects from cell death. J Virol.

[B30] Tiozzo R, Monti D, Straface E, Capri M, Croce MA, Rainaldi G, Franceschi C, Malorni W (1996). Antiproliferative activity of 3-aminobenzamide in A431 carcinoma cells is associated with a target effect on cytoskeleton. Biochem Biophys Res Commun.

[B31] Aviel S, Winberg G, Massucci M, Ciechanover A (2000). Degradation of the epstein-barr virus latent membrane protein 1 (LMP1) by the ubiquitin-proteasome pathway. Targeting via ubiquitination of the N-terminal residue. J Biol Chem.

[B32] Ullrich O, Reinheckel T, Sitte N, Hass R, Grune T, Davies KJ (1999). Poly-ADP ribose polymerase activates nuclear proteasome to degrade oxidatively damaged histones. Proc Natl Acad Sci USA.

[B33] Li Y, Urban JM, Cayer ML, Plummer HK, Heckman CA (2006). Actin-based features negatively regulated by protein kinase C-epsilon. Am J Physiol Cell Physiol.

[B34] Dawson CW, Tramountanis G, Eliopoulos AG, Young LS (2003). Epstein-Barr virus latent membrane protein 1 (LMP1) activates the phosphatidylinositol 3-kinase/Akt pathway to promote cell survival and induce actin filament remodeling. J Biol Chem.

[B35] Malorni W, Rivabene R, Straface E, Rainaldi G, Monti D, Salvioli S, Cossarizza A, Franceschi C (1995). 3-Aminobenzamide protects cells from UV-B-induced apoptosis by acting on cytoskeleton and substrate adhesion. Biochem Biophys Res Commun.

[B36] Poirier GG, de Murcia G, Jongstra-Bilen J, Niedergang C, Mandel P (1982). Poly(ADP-ribosyl)ation of polynucleosomes causes relaxation of chromatin structure. Proc Natl Acad Sci USA.

[B37] Klein G, Dombos L, Gothoskar B (1972). Sensitivity of Epstein-Barr virus (EBV) producer and non-producer human lymphoblastoid cell lines to superinfection with EB-virus. Int J Cancer.

[B38] Matusali G, Leo AD, Gavioli R, Bertelli L, Renzo LD, Mattia E (2007). Down-regulation of proteolytic complexes following EBV activation in BL cells. Biochem Biophys Res Commun.

[B39] Masciarelli S, Mattioli B, Galletti R, Samoggia P, Chichiarelli S, Mearini G, Mattia E (2002). Antisense to Epstein Barr Virus-encoded LMP1 does not affect the transcription of viral and cellular proliferation-related genes, but induces phenotypic effects on EBV-transformed B lymphocytes. Oncogene.

